# AD|ARC (Administrative Data | Agricultural Research Collection): Linking individual, household and farm business data for agricultural research – Challenges of linking agricultural datasets with individual-level records

**DOI:** 10.23889/ijpds.v8i2.2200

**Published:** 2023-09-14

**Authors:** Sian Morrison-Rees, Nicholas Webster, Paul Caskie

**Affiliations:** 1 Swansea University, Swansea, United Kingdom; 2 Welsh Government, Cardiff, United Kingdom; 3 Agri-food & Biosciences Institute, Belfast, United Kingdom

## Objectives

To create an anonymised research-ready data resource of farm households in the UK to generate evidence to support policy development, implementation and evaluation; improve understanding of farm family socio-economic characteristics; and assist stakeholders interested in understanding the health and well-being, resilience and prosperity and spatial properties of farming communities.

## Method

The ADARC research-ready data resource is being created in each nation of the UK. Each core dataset links agricultural datasets with individual- and household-level population data from the Census 2011. The farm business data is drawn from a number of sources (the Inter Departmental Business Register, EU Farm Structure Survey 2010 and Rural Payments data), which presented many data preparation challenges when linking to the Census of Population. Each core dataset will be linked to the health and education data available for that nation. Where possible, the ADARC datasets have been harmonised to allow federated querying across the UK.

## Results

The ADARC core datasets are complete in Wales and near completion in England and Scotland. Work is also well advanced in Northern Ireland. To the best of our knowledge, these are the first datasets linking agricultural data to individual- and household-level data at a population level. We will report the challenges experienced in linking farm business data with data at the household and individual level. This will include a description of the data preparation steps, the challenges encountered and solutions utilised at each stage of building this complex dataset from numerous, very different ‘parent’ datasets.

The structure and content of the core datasets will be presented as well as the potential benefits to researchers investigating the individual, household and community dimensions of agricultural research.

## Conclusion

Agriculture is currently facing a range of challenges with little known about the impact on farmers and farming households. ADARC introduces a new, powerful and versatile resource that will help inform debate and potentially lead to better outcomes on a range of issues relevant to farmers and farming communities.

